# Tumor-infiltrating lymphocytes for melanoma immunotherapy

**DOI:** 10.1080/2162402X.2023.2175506

**Published:** 2023-02-07

**Authors:** Oliver Kepp, Peng Liu, Laurence Zitvogel, Guido Kroemer

**Affiliations:** Centre de Recherche des Cordeliers, Equipe labellisée par la Ligue contre le cancer, Université Paris Cité, Sorbonne Université, Inserm U1138, Institut Universitaire de France, Paris, France; Metabolomics and Cell Biology Platforms, Institut Gustave Roussy, Villejuif, France; INSERM U1015, Equipe Labellisée - Ligue Nationale contre le Cancer, Villejuif, France; Gustave Roussy, ClinicObiome, Villejuif, France; Department of Biology, Institut du Cancer Paris CARPEM, Hôpital Européen Georges Pompidou, AP-HP, Paris, France

Oncological routine has incorporated the regular use of monoclonal antibodies targeting checkpoints of T cell function such as ipilimumab, which blocks cytotoxic T-lymphocyte–associated protein 4 (CTLA-4), or pembrolizumab and nivolumab, which both inhibit programmed cell death 1 (PD-1). Such immune checkpoint inhibitors have substantially improved recurrence-free survival (RFS) in patients with advanced stage melanoma.^1^ Moreover, the development of molecularly targeted therapies such as the BRAF inhibitor dabrafenib and the mitogen-activated protein inhibitor trametinib has extended treatment options for melanoma patients with advanced metastatic disease.^2^

Based on its survival benefits, ipilimumab was the first immune checkpoint blocker approved for the treatment of advanced melanoma.^3^ However, high-grade immune-related adverse effects are observed in 23% of patients.^4^ Moreover, in a large clinical trial enrolling 834 advanced melanoma patients, pembrolizumab was shown to cause prolonged progression-free and overall survival with less high-grade toxicity than ipilimumab.^4^ For this reason, currently, ipilimumab is used as second-line treatment in patients with metastatic melanoma, whereas PD-1 inhibitors alone or in combination with ipilimumab have been moved to first-line, inducing responses in 45% or 58% of patients, respectively. Nonetheless, double immune checkpoint blockade targeting PD-1 plus CTLA-4 is associated with a high incidence of severe adverse effects and is currently recommended primarily for patients with poor prognostic factors.

An activating mutation in the B-Raf proto-oncogene serine/threonine kinase (BRAF) gene (*BRAF*^V600E^) is present in more than 50% of melanoma patients. Thus, combination of dabrafenib plus trametinib is yet another treatment option for melanoma harboring such mutation. Although this therapy is associated with a high initial response rate, most patients develop resistance over time.^2^ Further combination approaches involving BRAF inhibition plus immune checkpoint blockade as well as the use of novel immune checkpoint blocking antibodies targeting lymphocyte-activation gene 3 (*LAG-3*) LAG3 have shown promising response rates. Thus, combination of anti-PD-1 and anti-LAG3 monoclonal antibodies has been associated with objective responses in 16% of patients with refractory disease but follow-up data are still missing.^5^ Nevertheless, as it stands the efficacy of both immune checkpoint inhibition or targeted approaches for patients with advanced stage melanoma remains limited and despite optimal treatment about half of the patients will eventually die from the disease.

Pioneered by Steven Rosenberg and colleagues, adoptive cell therapy with tumor-infiltrating lymphocytes (TILs) has been developed. This approach necessitates the *ex vivo* outgrowth and expansion of TILs, followed by their intravenous reinfusion into patients that have undergone preparative lymphodepletion by chemotherapy. The administration of recombinant human interleukin-2 (rhIL2) can be used to enhance the *in vivo* expansion of TILs. TIL therapy proofed safety and evidenced efficacy in phase 1–2 trials with 20% durable complete remission in patients with advanced stage melanoma and achieved objective responses in 36% of patients with advanced melanoma, including in anti-PD-1 refractory disease.^6–9^

Thus far, a direct comparison of TILs with standard immunotherapy has been missing. In a recent study published in the *The new England Journal of Medicine*, Rohaan et al. presented the results of a multicenter, open-label, randomized phase 3 clinical trial comparing ipilimumab with TIL therapy as first- or second-line treatment in patients with advanced unresectable stage IIIC or IV melanoma. A total of 168 patients, among whom 86% presented with disease refractory to anti–PD-1, were randomly assigned to TIL therapy or ipilimumab treatment.^5^

The median progression-free survival was 7.2 months (95% confidence interval [CI], 4.2–13.1) for patients receiving TIL therapy and 3.1 months (95% CI, 3.0–4.3) for those treated with ipilimumab. Objective response rate was 49% (95% CI, 38–60) and 21% (95% CI, 13–32) and median overall survival 25.8 months (95% CI, 18.2 to not reached) and 18.9 months (95% CI, 13.8–32.6) in TIL *versus* ipilimumab therapy, respectively. Of note, all patients who received TIL therapy experienced mainly chemotherapy-related high-grade adverse effects such as myelosuppression.^5^ Altogether, the trial led to the conclusions that non-cryopreserved, autologous TILs prepared from resected melanoma metastases by standard rapid expansion protocol are more efficient than ipilimumab in extending the progression-free survival of advanced melanoma patients.^5,10^

As a caveat, the complex logistics of cell therapies, as well as the limited availability of bioactive TILs, both may limit the general applicability of TIL therapy. Moreover, treatment-related adverse effects are frequent, requiring careful monitoring and management by the treating oncologist. Irrespective of these disadvantages, the results by Rohaan *et al*.^5^ underline the feasibility and utility of personalized autologous TIL therapy for advanced melanoma patients in specialized centers. Future studies must evaluate the optimization of TIL preparation as well as the combination of TIL therapy with targeted treatments or immunotherapies, including checkpoint inhibitors targeting CTLA-4 and the PD-1/PD-L1 interaction. Moreover, it will be important to further develop TIL-based therapies to other, more frequent malignancies than melanoma.^11–13^

## Data Availability

All data leading to the conclusion presented in this paper are annexed.

## References

[1] Galluzzi L, Vacchelli E, Bravo-San Pedro JM, Buque A, Senovilla L, Baracco EE, Bloy N, Castoldi F, Abastado JP, Agostinis P, et al. Classification of current anticancer immunotherapies. Oncotarget. 2014;5(24):12472–3. doi:10.18632/oncotarget.2998. 25537519 PMC4350348

[2] Eggermont AMM, Hamid O, Long GV, Luke JJ. Optimal systemic therapy for high-risk resectable melanoma. Nat Rev Clin Oncol. 2022;19(7):431–439. doi:10.1038/s41571-022-00630-4. 35468949 PMC11075933

[3] Sharma P, Allison JP. Immune checkpoint targeting in cancer therapy: toward combination strategies with curative potential. Cell. 2015;161(2):205–214. doi:10.1016/j.cell.2015.03.030. 25860605 PMC5905674

[4] Robert C, Schachter J, Long GV, Arance A, Grob JJ, Mortier L, Daud A, Carlino MS, McNeil C, Lotem M, et al. Pembrolizumab versus ipilimumab in advanced melanoma. N Engl J Med. 2015;372(26):2521–2532. doi:10.1056/NEJMoa1503093. 25891173

[5] Rohaan MW, Borch TH, van den Berg JH, Met O, Kessels R, Geukes Foppen MH, Stoltenborg Granhoj J, Nuijen B, Nijenhuis C, Jedema I, et al. Tumor-infiltrating lymphocyte therapy or ipilimumab in advanced melanoma. N Engl J Med. 2022;387(23):2113–2125. doi:10.1056/NEJMoa2210233. 36477031

[6] Rosenberg SA, Yang JC, Sherry RM, Kammula US, Hughes MS, Phan GQ, Citrin DE, Restifo NP, Robbins PF, Wunderlich JR, et al. Durable complete responses in heavily pretreated patients with metastatic melanoma using T-cell transfer immunotherapy. Clin Cancer Res. 2011;17(13):4550–4557. doi:10.1158/1078-0432.CCR-11-0116. 21498393 PMC3131487

[7] Fisher B, Packard BS, Read EJ, Carrasquillo JA, Carter CS, Topalian SL, Yang JC, Yolles P, Larson SM, Rosenberg SA. Tumor localization of adoptively transferred indium-111 labeled tumor infiltrating lymphocytes in patients with metastatic melanoma. J Clin Oncol. 1989;7:250–261. 2644399 10.1200/JCO.1989.7.2.250

[8] Topalian SL, Muul LM, Solomon D, Rosenberg SA. Expansion of human tumor infiltrating lymphocytes for use in immunotherapy trials. J Immunol Methods. 1987;102(1):127–141. doi:10.1016/S0022-1759(87)80018-2. 3305708

[9] Chandran SS, Somerville RPT, Yang JC, Sherry RM, Klebanoff CA, Goff SL, Wunderlich JR, Danforth DN, Zlott D, Paria BC, et al. Treatment of metastatic uveal melanoma with adoptive transfer of tumour-infiltrating lymphocytes: a single-centre, two-stage, single-arm, phase 2 study. Lancet Oncol. 2017;18(6):792–802. doi:10.1016/S1470-2045(17)30251-6. 28395880 PMC5490083

[10] Betof Warner A, Corrie PG, Hamid O. Tumor-infiltrating lymphocyte therapy in melanoma: facts to the future. Clin Cancer Res. 2022. doi:10.1158/1078-0432.CCR-22-1922. PMC10183807 36485001

[11] Knochelmann HM, Rivera-Reyes AM, Wyatt MM, Smith AS, Chamness R, Dwyer CJ, Bobian M, Rangel Rivera GO, Horton JD, Lilly M, et al. Modeling ex vivo tumor-infiltrating lymphocyte expansion from established solid malignancies. Oncoimmunology. 2021;10(1):1959101. doi:10.1080/2162402X.2021.1959101. 34408920 PMC8366547

[12] Lipp JJ, Wang L, Yang H, Yao F, Harrer N, Muller S, Berezowska S, Dorn P, Marti TM, Schmid RA, et al. Functional and molecular characterization of PD1(+) tumor-infiltrating lymphocytes from lung cancer patients. Oncoimmunology. 2022;11(1):2019466. doi:10.1080/2162402X.2021.2019466. 35154905 PMC8837234

[13] Weber EW, Maus MV, Mackall CL. The EMERGING landscape of immune cell therapies. Cell. 2020;181(1):46–62. doi:10.1016/j.cell.2020.03.001. 32243795 PMC8900215

